# Editorial: Bridging Scales and Levels

**DOI:** 10.1162/netn_e_00059

**Published:** 2018-09-01

**Authors:** Emma K. Towlson, Fabrizio De Vico Fallani

**Affiliations:** Center for Complex Network Research, Northeastern University, Boston, MA 02115, USA; Media Laboratory, Massachusetts Institute of Technology, Cambridge, MA 02139, USA; Inria, Aramis project-team, F-75013, Paris, France; Institut du Cerveau et de la Moelle épinière, ICM, Inserm U 1127, CNRS UMR 7225, Sorbonne Université, F-75013, Paris, France

## Abstract

Network neuroscience strives to understand the networks of the brain on all spatiotemporal scales and levels of observation. Current experimental and theoretical capabilities are beginning to facilitate a more holistic perspective, uniting these networks. This focus feature, “Bridging Scales and Levels,” aims to document current research and looks to future progress towards this vision.

One of the greatest assets of network neuroscience is its ability to transcend and unite disparate disciplinary approaches to understanding the brain. This is particularly valuable when it comes to spatiotemporal scales. From genes and proteins interacting in the cell, to signaling within neuronal populations, to the integration of cortical regions, and even the brain itself within the body, the brain is inescapably, on all scales, a network. Different timescales govern the operation of each part, from milliseconds-long molecular interactions, to the seconds and minutes for circuit and whole brain dynamics, to days and upwards for some brain-behavior and social interactions, and neurological diseases. At a given spatiotemporal scale, there is a further choice of the level on which to observe and question. These levels range from that of the single component (a gene of interest, the dynamics of a single neuron, or the role of a brain region), to circuitry and sub-systems, to the whole population. Ultimately, each of these pieces must be united to complete the puzzles presented by the brain. And that means finding ways to bridge the scales and levels.

The given scale and level often guides the type of approach taken, and the disciplinary specific knowledge and skills required. For instance, collecting fMRI data relies on imaging technology; data analysis may need expertise in signal processing, machine learning, statistics, etc. The scientific question and the outcome interpretation need crucial inputs from neuroscience-related disciplines. To piece all of these components together, there is thus a need within network neuroscience for forums within which these distinct communities can connect, share ideas, and learn to speak each other’s languages. This is what we set out to provide, when we held a one-day satellite symposium on Network Neuroscience at the international meeting NetSci 2017, in Indianapolis – the annual flagship conference for the Network Science Society. The strong attendance of the satellite, including around 120 participants, is testament to the enthusiasm within the network science community for neuroscientific applications and inspirations. Our ambitions necessarily included bridging communities further afield – welcoming in neuroscientific researchers who may not traditionally have attended a network science conference. This satellite was thus pivotal in that it marked the transition from the highly popular Brain Networks satellite that had run for the previous two years to a full-fledged Network Neuroscience satellite, enveloping all aspects of the field.

The result was a fast-paced, energetic, and vibrant day with representation from all corners of network neuroscience, via 17 oral presentations and nearly 40 posters. One talk was delivered remotely from Europe, and all talks were streamed via Facebook live (many of which obtained >200 views on the same day). Presenters were invited to submit their work to *Network Neuroscience*, to provide a snapshot of the topics of the day, and to ignite wider debate around the theme of this focus feature: “Bridging Scales and Levels.”

The relationship between structure and function remains of prominent interest in network neuroscience. Genetics-based approaches are gaining in popularity, and begin to attempt to bridge the very small spatial scale of genes with the structure and function of whole brain regions (Richiardi et al., 2015). A session on “Function and Dysfunction in the Human Brain” saw contributions exploring the nature of large-scale whole-brain connectivity when things work as expected (such as the nature of learning, Bassett and Mattar (2017)), and when they don’t (such as in Alzheimer’s disease, de Haan (2017)). On pages 306–322, Amico and Goñi (2018) extend an ICA-based framework to pick out concurrent features of structural and functional connectivity in connectomes from the Human Connecome Project.

30 years on from the seminal mapping of its connectome, *C. elegans* still commands a big presence in neuroscience (Yan et al., 2017). Indeed, given our unparalleled knowledge of its structure and behavior, and our experimental power to intervene, it is an organism with enormous potential to bridge the scales and levels. OpenWorm represents an international, interdisciplinary online community committed to creating a virtual worm. On pages 323–343, Olivares, Izquierdo, and Beer (2018) demonstrate, through computational simulations, that neurons in the ventral nerve cord are capable of producing a central pattern generator that drives locomotion.

Modern datasets are arriving with unprecedented, exquisite detail, and are often multimodal in nature, presenting opportunities to study scales and levels concurrently. There is naturally a demand for tools to study and integrate them. A “Computational Approaches” session included a hands-on data showcase demonstrating the Allen Institute for Brain Science’s latest data and tool releases. On pages 344–361, Keiriz et al. (2018) introduce a web-based visualization platform with which to explore brain networks for research and clinical purposes; the authors demonstrated their platform at the satellite with virtual reality headsets.

Looking to the future of network neuroscience, we must develop theoretical and experimental tools to link the scales and levels inherent in the brain. This is a challenge which necessarily includes bridging traditionally disparate research communities, and fostering an environment for effective communication and collaboration. The journal *Network Neuroscience* is one such vital avenue for researchers to share results and keep abreast of the newest advances. Academic meetings, including NetSci, are also starting to recognize this need, and have begun moving towards connecting the disjoint landscape. Much work remains to be done towards “Bridging Scales and Levels,” and the right forums to spark conversation and collaboration towards this goal are essential.
